# Causes of differences in the distribution of the invasive plants *Ambrosia artemisiifolia* and *Ambrosia trifida* in the Yili Valley, China

**DOI:** 10.1002/ece3.6902

**Published:** 2020-10-18

**Authors:** Hegan Dong, Zhanli Song, Tong Liu, Zhongquan Liu, Yan Liu, Baoxiong Chen, Qianqian Ma, Zhigang Li

**Affiliations:** ^1^ Xinjiang Production and Construction Corps Key Laboratory of Oasis Town and Mountain‐basin System Ecology Shihezi University Shihezi China; ^2^ College of Life Science Shihezi University Shihezi China; ^3^ Rural Energy and Environment Work Station in Yili Yining China; ^4^ Yili State Forestry Academy Yining China; ^5^ Yili Vocational and Technical College Yining China; ^6^ Rural Energy and Environment Agency Ministry of Agriculture and Rural Affairs of the People’s Republic of China Beijing China; ^7^ The First Affiliated Hospital of Shihezi University Medical College Shihezi China

## Abstract

*Ambrosia artemisiifolia* and *Ambrosia trifida* are two species of very harmful and invasive plants of the same genus. However, it remains unclear why *A. artemisiifolia* is more widely distributed than *A. trifida* worldwide. Distribution and abundance of these two species were surveyed and measured from 2010 to 2017 in the Yili Valley, Xinjiang, China. Soil temperature and humidity, main companion species, the biological characteristics in farmland ecotone, residential area, roadside and grassland, and water demand of the two species were determined and studied from 2017 to 2018. The area occupied by *A. artemisiifolia* in the Yili Valley was more extensive than that of *A. trifida*, while the abundance of *A. artemisiifolia* in grassland was less than that of *A. trifida* at eight years after invasion. The interspecific competitive ability of two species was stronger than those of companion species in farmland ecotone, residential, and roadside. In addition, *A. trifida* had greater interspecific competitive ability than other plant species in grassland. The seed size and seed weight of *A. trifida* were five times or eight times those of *A. artemisiifolia*. When comparing the changes under simulated annual precipitation of 840 mm versus 280 mm, the seed yield per m^2^ of *A. trifida* decreased from 50,185 to 19, while that of *A. artemisiifolia* decreased from 15,579 to 530.

## Graphical abstract

The differences in the distribution of the two species are mainly due to differences in interspecific competitive ability, seed size, and water dependence. The two species have stronger interspecific competitive ability than that of companion species, but *A. artemisiifolia* has a smaller seed size and stronger drought tolerance, which allows *A. artemisiifolia* to spread farther than *A. trifida*. The reason for wider distribution of *A. trifida* in grassland is that *A. trifida* has stronger interspecific competitive ability than *A. artemisiifolia* under sufficient water.

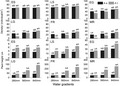

## 1 INTRODUCTION

Two invasive herbaceous species, *A. artemisiifolia* and *A. trifida*, have recently become troublesome weeds in several regions of the world, especially in central and eastern Europe as well as in China (Chrenová et al., 2010; Hamaoui‐Laguel et al., 2015; Kasprzyk et al., 2010; Qin et al., 2014). The existence of these two ragweed plants has greatly changed the biodiversity, structure, and function of the invaded ecosystems, seriously threatening agricultural production and human health (Hamaoui‐Laguel et al., 2015; Katz & Carey, 2014; Page & Nurse, 2015; Qin et al., 2014). Many reports have addressed the invasion process and distribution of *A. artemisiifolia* and *A. trifida* (Aikio et al., 2010; Bae et al., 2015; Chapman et al., 2016; Chauvel et al., 2006; Cunze et al., 2013; Joly et al., 2011; Leiblein‐Wild et al., 2016; Pinke et al., 2011; Richter et al., 2013; Skálová et al., 2017; Storkey et al., 2014). *Ambrosia artemisiifolia* and *A. trifida* belong to the same genus and originated in North America (Bazzaz, 1979; Essl et al., 2015). They spread to other continents as early as 1836 (Essl et al., 2015) or 1829 (Verloove, 2016) without considering its cultivation in botanical gardens. According to the global geographical distribution of the two species (CABI Invasive Species Compendium, https://www.cabi.org/isc/search/index?q=Ambrosia, accessed February 21, 2020), the distribution of *A. artemisiifolia* is more extensive than that of *A. trifida*. In addition, *A. artemisiifolia* and *A. trifida* occur in 80 and 40 countries, respectively (Montagnani et al., 2017).Why does *A. artemisiifolia* invade a larger area than *A. trifida* worldwide? In order to provide information necessary for the early warning of invasion by the two species, a more in‐depth study is necessary.

Differences in distribution between species are normally caused by differences in genetic adaptation to environmental conditions. *Ambrosia artemisiifolia* and *A. trifida* often invade roadsides, farmland ecotones, wastelands (Essl et al., 2009; Milakovic et al., 2014; Pinke et al., 2013), residential habitats(Ziska et al., 2003), and other disturbed areas (Bassett & Crompton, 1982; Essl et al., 2015; Fumanal et al., 2008; Milakovic et al., 2014). *Ambrosia artemisiifolia* is rarely found in grasslands (Bullock et al., 2012); however, *A. trifida* occurs in grasslands (Regnier et al., 2016). In terms of specific regions, there is partial overlap between the two species’ niches, but these two plants tend to invade different types of microhabitats. The main reason for the differences in habitat that they invade is not clear. Generally, invasive plant species have strong performance‐related traits, including those related to physiology, leaf‐area allocation, shoot allocation, growth rate, size, and fitness than do noninvasive plant species (van Kleunen et al., 2010, 2015). *Ambrosia artemisiifolia* and *A. trifida* both have relative strong interspecific competitive ability (Montagnani et al., 2017). The effect of interspecific competitive ability on the distribution differences of two species is unclear.

Water availability affects plant seed germination, growth, and reproduction, factors that are the basis of species distribution and competition, especially in arid and semiarid areas. Leiblein‐Wild and Lösch (2011) found that *A. artemisiifolia* grew well under moist soil conditions and that it can survive in dry soils. *Ambrosia trifida* needs more water than *A. artemisiifolia* (Abul‐Fatih & Bazzaz, 1979; Bassett & Crompton, 1982). It is not clear how the water use capacity affects the distribution difference of the two species. Moreover, the link between the differences in distribution and water demand of these two species during seed germination, plant growth, and reproduction period remains unclear.

Temperature has a significant effect on the distribution and growth of the two species (Pinke et al., 2011; Qin et al., 2014; Storkey et al., 2014). *Ambrosia artemisiifolia* and *A. trifida* have become widespread in temperate regions (Bassett & Crompton, 1975; Essl et al., 2015; Montagnani et al., 2017). Seeds of the two species require prolonged chilling to break dormancy (Bazzaz, 1979; Davis, 1930; Essl et al., 2015; Shrestha et al., 1999). Following seedling emergence, the rate of vegetative growth depends on temperature, but development occurs over a wide thermal range (Deen et al., 1998).

The Yili Valley, Xinjiang, China, covers an area of 56,400 km^2^ and contains a rich variety of habitats, including grasslands, farmlands, mountains, and residential areas (Jia et al., 2011). Our previous study found that *A. artemisiifolia* and *A. trifida* simultaneously invaded the same area of the Yili Valley in 2010, and we also found that the dominant habitat distributions of two species were different (Dong et al., 2017). Therefore, the Yili Valley provides a large, relatively closed field experiment site in which to study the beginning of an invasion by the two species along with their subsequent diffusion. This study can therefore help to explain the distribution differences and causes for successful invasion of *A. artemisiifolia* and *A. trifida*, providing insight into the reasons for the resulting distribution of these two species worldwide.

Distribution and abundance of *A. artemisiifolia* and *A. trifida* were surveyed and measured from 2010 to 2017 in the Yili Valley, Xinjiang, China. The soil physical and chemical properties, soil temperature and humidity, and the main companion species were determined in farmland ecotone, residential area, roadside, and grassland in 2017. Also, biological characteristics, such as density and coverage, plant height, number of seeds per plant, 100‐seed weight, and seed size of these two species and companion species (density and coverage, plant height) in four habitats, were measured in 2017. Moreover, the differences in water demand between the two species were studied through seed germination and garden experiments from October 2017 to October 2018. The following questions were explored: What were the differences in the distribution of these two species in the Yili Valley? What caused the differences in the distribution of these two species?

## 2 MATERIALS AND METHODS

### 2.1 Design of experiments

#### 2.1.1 Experiment 1: Distribution area and abundance of two Ambrosia species

##### Research area

The Yili Valley (42°14′–44°53′N, 80°09′–84°56′E) lies in the westernmost part of the Tianshan Mountain Range in the Xinjiang Autonomous Region. The region has an average annual temperature and precipitation of 10.4°C and 417.6 mm, respectively. Yili Valley can be thought of as a wet island in the arid area of Xinjiang, as it has an abundant and unique set of plant species, and the valley is listed as one of the five most important areas of terrestrial biodiversity in China (Chen, 1993).

Xinyuan County (43°03′–43°40′N, 82°28′–84°56′E) is located in the hinterland of the Gongnaisi grassland in the eastern part of the Yili Valley. This site is the main distribution area of *A. artemisiifolia* and *A. trifida*. The average annual temperature and precipitation are 8.1°C and 480 mm, respectively. We studied the interspecific competitive ability, seed size, and water demand differences between the two species in farmland ecotone, residential area, roadside, and grassland in Xinyuan County, because these four habitats were the main distribution areas of the two species (Dong et al., 2017), and there were relatively large differences in water status, temperature status, and companion species between those four habitats.

##### Distribution area

The distribution areas of *A. artemisiifolia* and *A. trifida* in the Yili Valley were surveyed and measured during the growth periods from 2010 to 2017. Every year, through a large number of field censuses, new distributional points of the two species were recorded with GPS to determine the current distribution boundaries of the two species. In order to ensure the accuracy of the measurements, we included a sufficient number of boundary points, with the distances between two consecutive points limited to 2–3 km. These points were then marked on a Google map to calculate the distribution areas.

##### Abundance

In July 2010, we set an observation point every 1.5 km along National Road 218 from Zeketai Town (43°37′–43°40′N, 83°10′–83°39′E) to Nalati Town (43°15′–43°37′N, 83°85′–84°56′E) in Xinyuan County; observation points were laid out within 0–10 km on both sides of the road, and each observation point covered 10 m × 10 m. The observation points included farmland ecotones, residential areas, grasslands, and roadside habitats. There were 25 points in each habitat (100 points in total). From 2010 to 2017, we investigated the incidence of the two *Ambrosia* species and calculated their distribution and abundance in each plot using Equations (1) and (2), respectively:

(1) Abundancein habit=(numberofoccurrencesinahabitat/25)×100%.

(2) Total abundance=(numberofoccurrencesinallhabitats/100)×100%.

#### 2.1.2 Experiment 2: Soil physical and chemical properties, soil temperature and humidity, and companion species in four habitats

##### Soil physical and chemical properties

The differences in soil physical and chemical properties were compared between farmland ecotones, residential areas, grasslands, and roadsides. The upper 0–20 cm of soil from four habitats (selected at observation points determined in 2010 in Experiment 1 where *A. artemisiifolia* and *A. trifida* were present) was divided into two layers. The soil in each 10‐cm layer was sampled, and soil properties were determined in July 2017 as follows. Total nitrogen, total phosphorus, and total potassium were determined using the micro‐Kjeldahl, sodium hydroxide melting‐molybdenum anticolorimetric, and flame photometry methods, respectively. Soil pH was measured using a Mettler‐Toledo pH meter (UB‐10, USA), and soil conductivity was measured using a conductivity meter (Hach, USA). Soil organic matter content was checked using the K_2_CrO_7_‐H_2_SO_4_ external heating method. Alkaline hydrolysis nitrogen, available P, and available K were measured using the alkaline hydrolysis diffusion method, Mo‐Sb colorimetry, and the ammonium acetate method, respectively. Soil samples from each habitat were taken three times in three individual sites (more than 5 km apart), and a total of 3 (repetition) × 4 (habitat) × 2 (soil layer), which resulted in 24 samples being collected.

##### Soil temperature and humidity

In order to compare the water demand between the two species analyzed, soil temperature and humidity meters (Watch Dog 1200, USA) were placed in the 10‐cm soil layer in the four habitats on 1 September 2017; the meters were removed on 2 October 2018. Each temperature and humidity meter recorded data every hour. The data from 1 October 2017 to 30 September 2018 were used to analyze the annual conditions. Three temperature and humidity recorders were placed in three individual sites for each habitat, and a total of 3 (repetition) × 4 (habitat) = 12 recording units were set up.

The data for temperature and humidity were divided into four parts, namely the winter season (1 October 2017–31 March 2018; WP), seedling period (1 April –31 May 2018; SP), growing period (1 June –31 July 2018; GP), and flowering and fruiting period (1 August –30 September 2018; FFP). The average temperature and humidity data for each period were calculated.

##### Companion species

In the early seedling (April 20; ES) period, late seedling (May 20; LS) period, early growth (June 20; EG) period, late growth (July 20; LG) period, flowering (August 20; FR) period, and maturity (September 20; MR) period in 2017, the main companion species were counted in the four habitats.

#### 2.1.3 Experiment 3: Observation of biological characteristics

In the ES (April 20), LS (May 20), EG (June 20), LG (July 20), FR (August 20), and MR (September 20) periods in 2017, the densities, coverage, and plant heights of the two *Ambrosia* species and companion species were measured in the four habitats. Each observation plot was 5 m × 5 m. The plant heights were measured for 30 plants of *A. artemisiifolia* and *A. trifida*, and 30 plants of companion species in each plot. If there were fewer than 30 *Ambrosia* or companion species plants, we measure all of them. Three plots from each habitat were taken in three individual sites, for a total of 3 (repetition) × 4 (habitat) sampling units in 12 plots being set up.

Six *A. artemisiifolia* and *A. trifida* plants were randomly selected from each sample plot, and all seeds counted on these plants were removed in September 2017. If some seeds had fallen, we estimated the number based on the locations of the seeds. A total of 100 seeds from each plant were randomly selected, air‐dried, and weighed with 0.0001 g precision on an electronic balance (BDS, China). Twenty seeds were randomly selected from each plant, and the lengths and widths of these seeds were measured with Vernier calipers (BDS, China) to calculate the average seed size using Equations (3) and (4), with three repetitions for each:

(3) Seedsize=seedlength×seedwidth.

(4) $$\text{Seed}\;\text{yield}\;m^{‐2}\;=\;\text{average}\;\text{number}\;\text{of}\;\text{seeds}\;\text{per}\;\text{plants}\;\times \;\text{the}\;\text{number}\;\text{of}\;\text{plants}\;m^{‐2}.$$

#### 2.1.4 Experiment 4: Water demand differences between two Ambrosia species

##### Seed germination

Seed germination was analyzed in the laboratory. In October 2016, the seeds of *A. artemisiifolia* and *A. trifida* were collected from four habitats in the Yili Valley and combined. The amount of seeds between habitats was set to be equal, and the seeds were initially stored in the dark at 0–5°C in a cold storage room with 40% relative humidity (Bae et al., 2016). In June 2017, 50 g heat‐dried in situ soil samples were weighed, and each sample was placed in a Petri dish. Next, 2.5, 5, 7.5, 10, and 12.5 g of distilled water was added to each Petri dish, resulting in the soil moisture contents in the various Petri dishes of 5%, 10%, 15%, 20%, and 25%, respectively. For each sample, 20 fully developed undamaged same‐sized seeds of *A. artemisiifolia* or *A. trifida* were uniformly spread on the soil surface in Petri dishes. Each group of seeds was evenly placed in Petri dishes. The seeds were treated in a climatic chamber (GTOP‐150Y, China) for 60 days at 20–10°C, 12‐hr/12‐hr light/darkness, and 3,000 lx light intensity, after which the germination rate was calculated by counting the number of germinated seeds. Seeds with the seed radicle at least 0.2 mm long were considered to have germinated. Seed germination was checked every day. When no seeds germinated in a single Petri dish for five consecutive days, it was regarded as the end of germination.

##### Growth and reproduction

A plant growth experiment was performed in the experimental garden from October 2017 to October 2018 in Yining City (43°50′–44°09′N,80°04′–81°29′E), located in the Yili Valley. This locale has an average annual temperature of 10.5°C and an average annual precipitation of 280 mm. Three irrigation treatment gradients were established in the experimental garden: (1) no irrigation with 280 mm of annual precipitation; (2) 2,800 m^3^/hm^2^ of irrigation during the growth period (equivalent to 560 mm of annual precipitation supplemented by 400 m^3^/hm^2^ of irrigation every month from April to October 2018), and (3) 5,600 m^3^/hm^2^ of irrigation during growth period (equivalent to 840 mm of annual precipitation supplemented by 800 m^3^/hm^2^ of irrigation every month from April to October 2018). Each water treatment was tested with three plots, and eighteen plots were randomly arranged with 3 m × 3 m plots for each irrigation sample area. Plastic film was buried vertically to a depth of 40 cm in the soil around each irrigation plot to separate the water received in each plot. Each plot was uniformly sprinkled with 900 seeds of *A. artemisiifolia* or *A. trifida*.

##### Data collection

Density and plant height were observed during the ES (April 15), LS (May 15), EG (June 15), LG (July 15), FR (August 15), and MR (September 20) periods in 2018, and seed yield per m^2^ was observed in MR (September 20) in 2018. The statistical analysis of density, plant height, and seed yield was the same as in Experiment 3.

#### 2.1.5 Statistical analysis methods

One‐way analysis of variance (ANOVA) and multiple least significant difference comparisons were used to explore the differences in soil physical and chemical properties (Table 1) and soil temperature and humidity (Figure 3) between the four habitats, while 100‐seed weight, seed size, number of seeds per plant, and seed yield per m^2^ were compared between *A. artemisiifolia* and *A. trifida* (Figure 5). ANOVA was also used to examine differences in densities, coverage, and plant heights of *A. artemisiifolia*, *A. trifida*, and companion species between the four habitats (Figure 4). ANOVA, multiple least significant difference comparisons, and *t* tests were used to explore the differences in seed germination (Table 3), density and plant height (Figure 6), and seed yield (Table 4) of *A. artemisiifolia* and *A. trifida* in different water gradients.IBM SPSS Statistics 20 was used for data analysis, and OriginPro 8.5 was employed for graphics.

#### 2.1.6 Results

### 2.2 Distribution differences between *A. artemisiifolia* and *A. trifida* (Experiment 1)

**Figure 1:**
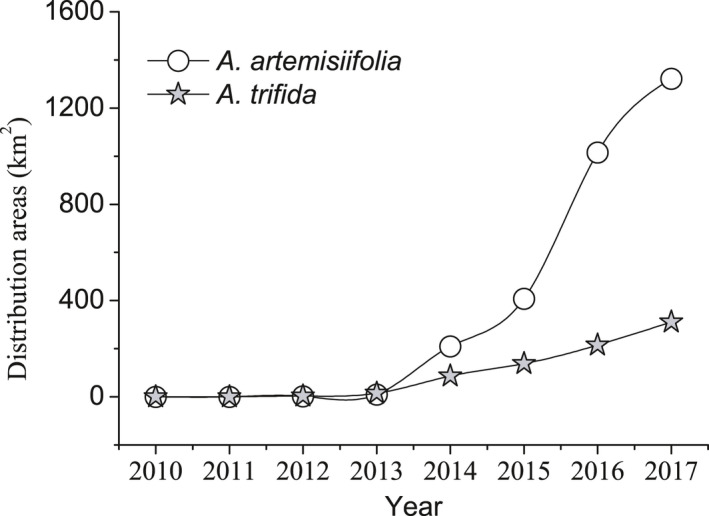
Total area occupied by *Ambrosia trifida* and *Ambrosia trifida* in the Yili Valley from 2010 to 2017

*Ambrosia artemisiifolia* and *A. trifida* invaded the Yili Valley starting in 2010. Since 2014, the areas occupied by these two species have increased rapidly, although *A. artemisiifolia* is distributed over a larger area than *A. trifida*. By 2017, these two species had occupied 1,322 and 311 km^2^, respectively; thus, the former occupied 4.25 times the area inhabited by *A. trifida* (Figure 1).

**Figure 2:**
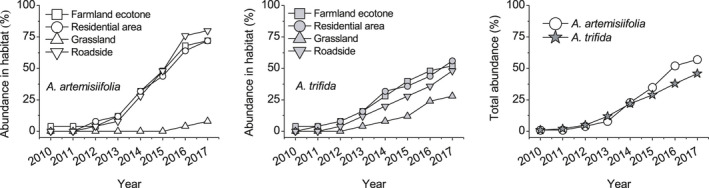
Abundance of *Ambrosia artemisiifolia* and *Ambrosia trifida* in occupied areas of the Yili Valley from 2010 to 2017

The species abundances were measured in the 25 plots in each habitat. From 2010 to 2017, the abundance of *A. artemisiifolia* was higher than that of *A. trifida* and increased rapidly in farmland ecotone, residential area, and roadside habitats. By 2017, total abundance of *A. artemisiifolia* and *A. trifida* was 57% and 39%, respectively, so that *A. artemisiifolia* was 1.46 times more abundant than *A. trifida*. However, the abundance of *A. artemisiifolia* in grassland was less than that of *A. trifida*, where the latter was 3.5 times more abundant than the former (Figure 2).

### 2.3 Soil physical and chemical properties, soil temperature and humidity, and companion species in four habitats (Experiment 2)

Soil physical and chemical properties showed little difference among different habitats; however, the soil total nitrogen levels in farmland ecotones and roadsides were higher and lower, respectively, than those in other habitats. The contents of available phosphorus and available potassium in grasslands were lower than those in other habitats. Soil organic matter content in grasslands was higher than that in other habitats (Table 1).

**Table 1:** Soil physical and chemical properties in four habitats in the Yili Valley

Index	Soil depth (cm)	Farmland ecotone	Residential area	Grassland	Roadside
Total nitrogen	0–10	17.4 ± 2.33a	9.82 ± 1.13b	13.3 ± 2.01b	5.96 ± 0.41c
(%)	10–20	14.1 ± 2.11a	9.2 ± 1.78b	9.63 ± 1.67b	5.27 ± 0.67c
Total phosphorus	0–10	0.0766 ± 0.0042b	0.0865 ± 0.0066b	0.0768 ± 0.0067b	0.135 ± 0.0092a
(g/kg)	10–20	0.122 ± 0.0246a	0.157 ± 0.0212a	0.111 ± 0.014a	0.123 ± 0.0111a
Total potassium	0–10	21.8 ± 3.85a	24.6 ± 5.46a	22.3 ± 4.27a	20.7 ± 4.11a
(g/kg)	10–20	26 ± 5.81a	24.4 ± 6.17a	24.9 ± 6.21a	21.6 ± 2.17a
Available nitrogen	0–10	4.35 ± 1.21a	3.71 ± 0.81a	2 ± 0.66a	2.98 ± 0.39a
(mg/kg)	10–20	4.02 ± 0.95a	2.6 ± 0.38a	1.9 ± 0.46a	2.86 ± 0.43a
Available phosphorus	0–10	0.185 ± 0.042a	0.152 ± 0.021a	0.0631 ± 0.009b	0.144 ± 0.012a
(mg/kg)	10–20	0.141 ± 0.011a	0.117 ± 0.021a	0.0285 ± 0.0031b	0.151 ± 0.018a
Available potassium	0–10	95.4 ± 12.2a	78.1 ± 6.71b	14.4 ± 2.62c	42.7 ± 6.78b
(mg/kg)	10–20	88.3 ± 10.3a	43.1 ± 5.12b	11.9 ± 1.97c	40.9 ± 8.29b
Organic matter	0–10	8.21 ± 1.33b	11.3 ± 2.11b	16.9 ± 2.62a	9.17 ± 0.77b
(%)	10–20	9.16 ± 1.31b	10.2 ± 1.41b	16.9 ± 1.27a	6.15 ± 0.78b
pH	0–10	7.44 ± 0.56a	7.55 ± 0.86a	7.93 ± 0.93a	7.63 ± 0.44a
	10–20	7.54 ± 0.64a	7.61 ± 0.51a	7.98 ± 0.62a	7.72 ± 0.41a
Conductivity	0–10	12.7 ± 0.34a	12.2 ± 0.51a	11.9 ± 0.17a	12.7 ± 0.27a
(μs/m)	10–20	12.1 ± 0.26a	12.1 ± 0.37a	11.8 ± 0.42a	12.7 ± 0.39a

Note

Different letters indicate significant differences at *p* < .05 using least significant difference tests for different habitats.

**Figure 3:**
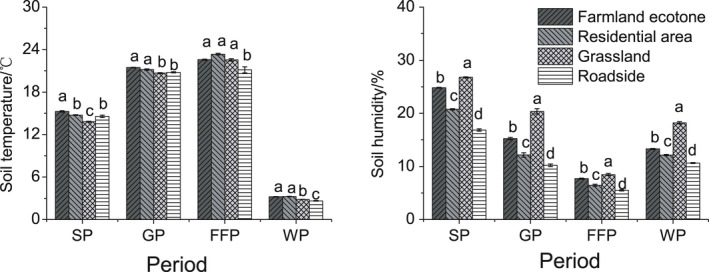
Soil temperature and humidity in the seedling period (SP), growing season (GP), flowering and fruiting period (FFP), and winter season (WP) of the four habitats analyzed in the present study in the Yili Valley. Different letters indicate significant differences at *p* < .05 using least significant difference tests for different habitats

In SP, the soil temperature of farmland was significantly higher than in other habitats, and that of grassland was significantly lower than in other habitats; in GP, the soil temperatures of grassland and roadside were significantly lower than those of other habitats; in FFP, the roadside temperature was significantly lower than those of other habitats. The soil moisture in different habitats showed significant differences in different periods, and the values of soil moisture were ranked as follows: grassland > farmland ecotone > residential area > roadside (Figure 3).

The types of companion species in farmland ecotones, residential areas, and roadsides were similar and quite different from those of grassland (Table 2).

**Table 2:** Main companion species in four habitats in the Yili Valley

Habitats	Companion species
Farmland ecotone	*Arctium lappa* L., *Amaranthus retroflexus* L., *Artemisia annua* L., *Chenopodium album* L., *Setaria viridis* L., *Urtica fissa* E. Pritz., *Cannabis sativa* L., *Portulaca oleracea* L., *Convolvulus arvensis* L., *Datura stramonium* L., *Iris lactea* Pall. var. *chinensis* (Fisch.) Koidz., *Artemisia argyi* Levl. et Vant., *Bromus inermis* Layss., *Avena sativa* L., *Polygonum hydropiper* L., *Capsella bursa‐pastoris* L., *Daucus carota* L., *Echinochloa crus‐galli* (L.) Beauv., and *Cichorium intybus* L.
Residential area	*Arctium lappa* L., *Amaranthus retroflexus* L., *Artemisia annua* L., *Chenopodium album* L., *Setaria viridis* L., *Urtica fissa* E. Pritz., *Cannabis sativa* L., *Portulaca oleracea* L., *Convolvulus arvensis* L., *Datura stramonium* L., *Iris lactea* Pall. var. *chinensis* (Fisch.) Koidz., *Artemisia argyi* Levl. et Vant., *Avena sativa* L., *Polygonum hydropiper* L., *Capsella bursa‐pastoris* L., *Daucus carota* L., *Echinochloa crus‐galli* (L.) Beauv., *Cichorium intybus* L., and *Plantago lanceolata* L.
Grassland	*Achillea millefolium* L., *Cirsium setosum*, *Sonchus oleraceus* L., *Agrostis matsumurae* Hack. ex Honda, *Cichorium intybus* L., *Agrimonia pilosa* L., *Conyza Canadensis* L., *Thalictrum aquilegiifolium* L., *Daucus carota* L., *Sophora alopecuroides* L, *Trifolium repens* L., *Urtica fissa* E. Pritz., *Cannabis sativa* L., *Amaranthus retroflexus* L., *Impatiens brachycentra* Kar. et Kir.
Roadside	*Arctium lappa* L., *Amaranthus retroflexus* L., *Artemisia annua* L., *Chenopodium album* L., *Setaria viridis* L., *Urtica fissa* E. Pritz., *Cannabis sativa* L., *Portulaca oleracea* L., *Convolvulus arvensis* L., *Datura stramonium* L., *Iris lactea* Pall. var. *chinensis* (Fisch.) Koidz., *Artemisia argyi* Levl. et Vant., *Bromus inermis* Layss., *Avena sativa* L., *Polygonum hydropiper* L., *Capsella bursa‐pastoris* L., *Daucus carota* L., *Cichorium intybus* L., and *Plantago lanceolata* L.

Note

We used nomenclature of Linnaeus.

### 2.4 Biological characteristics of *A. artemisiifolia, A. trifida* and companion species (Experiment 3)

**Figure 4:**
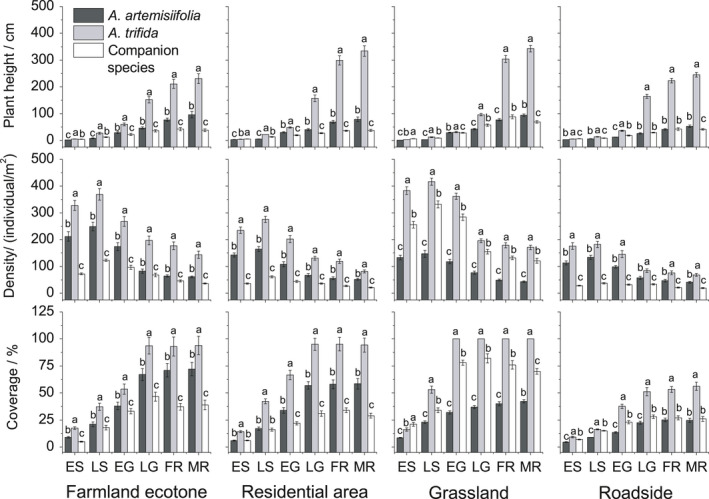
The density and coverage as well as plant height in the early seedling (ES) period, late seedling (LS) period, early growth (EG) period, late growth (LG) period, flowering (FR) period, and maturity (MR) period of *Ambrosia artemisiifolia*, *Ambrosia trifida*, and companion species in the four habitats in the Yili Valley. Different letters indicate significant differences at *p* < .05 using least significant difference tests for *Ambrosia artemisiifolia*, *Ambrosia trifida*, and companion species

*Ambrosia trifida* was significantly taller than other plant species in all habitats from LS period to MR period, being 3.45–8.3 times taller than the companion species in the FR period. *Ambrosia artemisiifolia* was significantly taller than companion species in the farmland ecotone and residential area from ES period to MR period (Figure 4).

The density of *A. trifida* was significantly higher than that of the other species in all habitats, reaching 1.35–4.4 times that of the companion species in the FR period. The density of *A. artemisiifolia* was higher than that of the companion species in the farmland ecotone, residential area, and roadside, at 1.39–2.23 times that of the companion species in FR period. However, the density of *A. artemisiifolia* was lower than that of the companion species in grassland, at only 0.37 times the density of the companion species in FR period (Figure 4).

The coverage of *A. trifida* was significantly greater than that of the other species in all habitats from LS period to MR period, at 1.31–2.8 times that of the companion species in FR period, respectively. The coverage of *A. artemisiifolia* was significantly higher than that of the companion species in the farmland ecotone and residential area from EG period to MR period, at 1.84 and 1.7 times that of the companion species in FR period, respectively. However, the coverage was significantly lower than the companion species in grassland, at 0.53 times that of the companion species in FR period (Figure 4).

**Figure 5:**
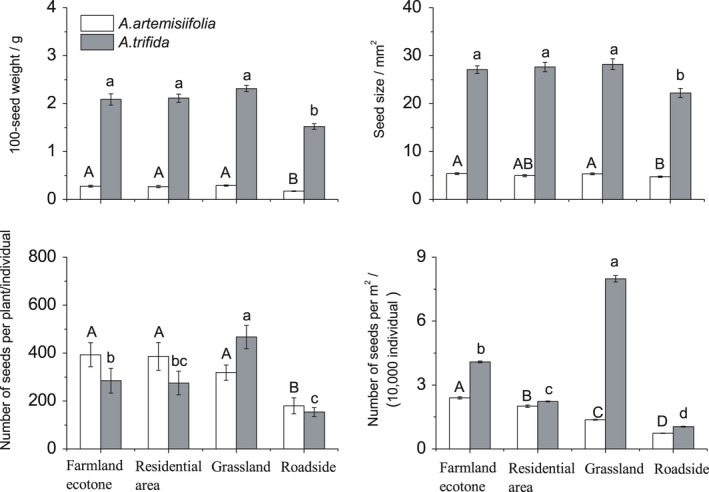
The 100‐seed weight, seed size, number of seeds per plant, and number of seeds per m2 of *Ambrosia artemisiifolia* and *Ambrosia trifida* in the four habitats in the Yili Valley. Different letters indicate significant differences at *p* < .05 using least significant difference tests for different habitats. Capital and lowercase letters are used for *Ambrosia artemisiifolia* and *Ambrosia trifida*, respectively

The 100‐seed weight and seed size of *A. artemisiifolia* and *A. trifida* in roadside habitats were significantly lower than those in other habitats. The seed size of *A. trifida* was about five times that of *A. artemisiifolia*, while the weight of *A. trifida* seeds was about eight times that of *A. artemisiifolia* (Figure 5).

The numbers of seeds per plant of *A. artemisiifolia* and *A. trifida* in roadside habitat were significantly lower than in other habitats. *Ambrosia trifida* produced a significantly greater number of seeds per plant in grassland than in other habitats. Meanwhile, *A. artemisiifolia* produced fewer seeds per plant than *A. trifida* in grassland, but more than *A. trifida* in other habitats (Figure 5).

Seed yield per m^2^ of *A. artemisiifolia* in various habitats was ranked as follows: farmland ecotone >residential area >grassland >roadside. Seed yield per m^2^ of *A. trifida* in various habitats differed as follows: grassland >farmland ecotone > residential area >roadside. Seed yield per m^2^ of *A. artemisiifolia* was less than that of *A. trifida* in all habitats (Figure 5).

### 2.5 The water demand for seed germination, plant growth, and reproduction in *A. artemisiifolia* and *A. trifida* (Experiment 4)

The seed germination rates of *A. artemisiifolia* and *A. trifida* increased with increasing soil moisture. However, no significant difference in seed germination rate was observed when comparing these two species under the same soil moisture content (Table 3).

**Table 3:** Seed germination of *Ambrosia artemisiifolia (A. a.)* and *Ambrosia trifida (A. t.)* in different soil moisture contents

Soil moisture contents (%)	5	10	15	20	25
Seed germination of *A. a*. (%)	0cA	51.7 ± 2.6bA	75.6 ± 3.2aA	78.3 ± 2.9aA	82.2 ± 2.8aA
Seed germination of *A. t*. (%)	0cA	62.2 ± 4.4bA	76.7 ± 3.3aA	81.7 ± 4.5aA	85.0 ± 2.9aA

Note

Different capital letters indicate significant differences at *p* < .05 using independent *t* tests for *A. artemisiifolia* and *A. trifida*. Different lowercase letters indicate significant differences at *p* < .05 using least significant difference tests for different soil moisture contents.

**Figure 6:**
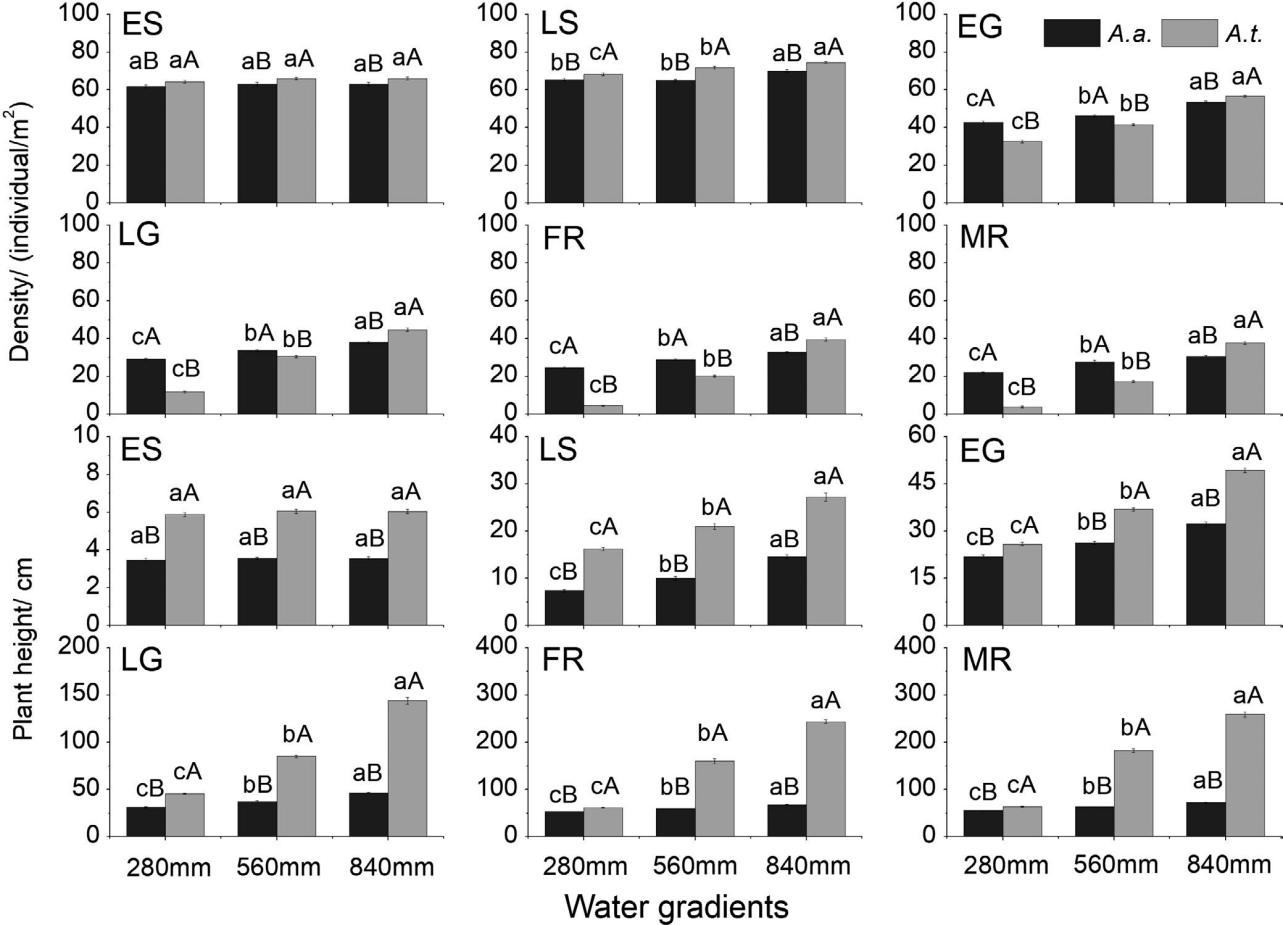
The density and plant height in the early seedling (ES) period, late seedling (LS) period, early growth (EG) period, late growth (LG) period, flowering (FR) period, and maturity (MR) period in 280 mm, 560 mm and 840 mm of simulated annual precipitation for *Ambrosia artemisiifolia* (*A. a*) and *Ambrosia trifida* (*A. t*). Different capital letters indicate significant differences at *p* < .05 using independent *t* tests for *A. artemisiifolia* and *A. trifida*. Different lowercase letters indicate significant differences at *p* < .05 using least significant difference tests for different water gradients

Under 560 and 840 mm of simulated annual precipitation, *A. artemisiifolia* and *A. trifida* both grew better than under 280 mm of annual precipitation during the growing period. The growth of *A. trifida* was very poor, with a low seed yield, whereas *A. artemisiifolia* grew better than *A. trifida* under 280 mm of annual precipitation. When comparing plants experiencing 840 mm of simulated rainfall and 280 mm of annual precipitation during the growing period, the densities and plant heights of these two species were not significantly different in ES. From the FR to MR, the density, plant height, and seed yield of *A. trifida* decreased more than those of *A. artemisiifolia*. In FR, the density and plant height of *A. trifida* decreased by 88.5% and 74.5%, respectively, while those of *A. artemisiifolia* decreased by 24.5% and 21.6%, respectively (Figure 6). The seed yield per m^2^ of *A. trifida* decreased from 50,185 to 19, while that of *A. artemisiifolia* decreased from 15,579 to 530 (Table 4).

**Table 4:** Seed yield of *Ambrosia artemisiifolia (A. a.)* and *Ambrosia trifida (A. t.)* under 280 mm, 560 mm and 840 mm of simulated annual precipitation

Water gradient	Seed yield/(individual/m^2^)	
	*A. a*.	*A. t*.
280 mm	530 ± 37.2cA	19.1 ± 1.46cB
560 mm	4,217 ± 229bA	4,661 ± 139bB
840 mm	15,579 ± 698aB	50,185 ± 2652aA

Note

Different capital letters indicate significant differences at *p* < .05 using independent *t* tests for *A. artemisiifolia* and *A. trifida*. Different lowercase letters indicate significant differences at *p* < .05 using least significant difference tests for different water gradients.

## 3 DISCUSSION

*Ambrosia artemisiifolia* invaded more quickly than *A. trifida* in the Yili Valley. In roadside, farmland ecotone, and residential area habitats with relatively poor water availability and weak interspecific competition, *A. artemisiifolia* was much more abundant than *A. trifida*. In grassland with relatively rich water availability and strong interspecific competition, *A. trifida* was much more abundant than *A. artemisiifolia* (Figure 2). In the study area, more types and larger areas of suitable habitat are available to *A. artemisiifolia* than to *A. trifida*, which is consistent with the distribution of these two species worldwide (Bullock et al., 2012; Chauvel et al., 2006; Follak et al., 2013; Montagnani et al., 2017; Regnier et al., 2016).

Greater population density, higher plant height, and greater coverage are conducive to successful plant invasion (Chapman et al., 2014, 2016). Although the density, height, and coverage of *A. trifida* were higher than those of *A. artemisiifolia* and companion species in roadside, farmland ecotone, and residential area in the present study (Figure 4), the distribution points of *A. trifida* were all located in low‐lying and waterlogged areas (Figure 2). *Ambrosia artemisiifolia* is highly competitive in continuously disturbed habitats such as roadsides and farmland ecotones (Bullock et al., 2012; Gentili et al., 2015, 2017; Kazinczi et al., 2008; Novak et al., 2009) as the disturbances decrease competition. *Ambrosia trifida* is widely distributed in grassland as the density, height, and coverage of *A. trifida* are higher than those of *A. artemisiifolia* and companion species (Figure 4). The life‐history strategy of *A. trifida* is mostly based on rapid growth that allows the plants to quickly reach a greater height and biomass than other plants (Abul‐Fatih & Bazzaz, 1979). Stronger interspecific competitive ability of *A. trifida* may explain larger distribution of the species in grassland.

The primary means of dispersal of *A. artemisiifolia* and *A. trifida* seeds are barochory (Basset & Crompton, 1975; Montagnaniet al., 2017).The medium‐distance and long‐distance dispersal of *A. artemisiifolia* and *A. trifida* is driven by human activities and obstruction in many ways (Bullock et al., 2012). Seed size is an important factor affecting seed diffusion and species distribution (Washitani & Nishiyama, 1992). *Ambrosia artemisiifolia* has lighter and smaller seeds (Figure 5), so *A. artemisiifolia* seeds are easier to spread in habitats with more human activity such as residential area and roadside (Bullock et al., 2012; Essl et al., 2009; Skálová et al., 2017). Easier spread of seeds of *A. artemisiifolia* may explain larger distribution of the species in the Yili Valley. In addition, the long‐term seed bank of *A. artemisiifolia* (Fumanal et al., 2008; Webster et al., 2003) may be mentioned as a factor stabilizing populations, especially in very dry years when seed production is low.

*Ambrosia artemisiifolia* can grow well and produce more seeds than *A. trifida* with a limited water supply when the latter produces almost no seeds (Table 4). This shows that *A. artemisiifolia* has a stronger ability than *A. trifida* to tolerate drought. The net photosynthetic rate of *A. artemisiifolia* decreases during periods of reduced soil water content (Bazzaz, 1973), but the plants recover rapidly from short‐term droughts (Bazzaz, 1973, 1974). In unusually dry years or on dry sites, *A. artemisiifolia* plants have stunted growth but remain able to produce seeds, albeit in small quantities (Leiblein‐Wild & Lösch, 2011; Raynal & Bazzaz, 1975). Stronger drought tolerance of *A. artemisiifolia* may explain larger distribution of the species in roadside, farmland ecotone, and residential area habitats with relatively poor water availability. Low rainfall is a limiting factor for the growth of *A. trifida* (Basset & Crompton, 1982). Therefore, *A. trifida* can invasion success only when adequate water is available.

*Ambrosia artemisiifolia* and *A. trifida* were mainly distributed in farmland ecotone, roadside, residential area, grassland valley, and other accumulated water in the Yili Valley (Dong et al., 2013), and there was no obvious law for the difference in soil temperature of the four habitats in different periods (Figure 3). Therefore, we believe that the existing distribution pattern of the two species is not mainly affected by temperature in the Yili Valley.

Since the causes of species distribution include factors other than interspecific competition, seed size, and water demand, other issues need to be discussed in future work if researchers wish to better explain the reasons for the differences between these two species. Additional factors to investigate include the following: (a) How temperature and water work collectively to affect the germination, growth, and reproduction of these two species; and (b) quantitative analysis of the influence of the difference in seed size on the difference in distribution of the two species.

## CONFLICT OF INTEREST

None declared.

## AUTHOR CONTRIBUTION

**Hegan dong:** Conceptualization (lead); Data curation (lead); Formal analysis (lead); Funding acquisition (lead); Investigation (lead); Methodology (lead); Project administration (lead); Resources (lead); Software (lead); Supervision (lead); Validation (lead); Visualization (lead); Writing‐original draft (lead); Writing‐review & editing (lead). **Zhanli Song:** Formal analysis (equal); Investigation (equal); Writing‐original draft (equal); Writing‐review & editing (equal). **Tong Liu:** Conceptualization (lead); Data curation (equal); Formal analysis (equal); Funding acquisition (lead); Project administration (lead); Resources (equal); Writing‐original draft (equal); Writing‐review & editing (equal). **Zhongquan Liu:** Data curation (equal); Formal analysis (equal); Investigation (equal); Methodology (equal); Software (equal); Writing‐original draft (equal). **Yan Liu:** Formal analysis (equal); Investigation (equal); Resources (equal); Writing‐original draft (equal). **Baoxiong Chen:** Data curation (equal); Methodology (equal). **Qianqian Ma:** Formal analysis (equal); Software (equal). **Zhigang Li:** Software (equal).

## DATA AVAILABILITY STATEMENT

All raw data have been uploaded to Dryad with DOI accession number: https://datadryad.org/stash/dataset/doi:10.5061/dryad.fttdz08p5.
